# *NSUN2* rs13181449 variant decreases the risk of oral cancer development

**DOI:** 10.7150/ijms.113676

**Published:** 2025-06-20

**Authors:** Li-Chung Hung, Cheng-Chen Huang, Yen-Ting Lu, Chun-Wen Su, Chiao-Wen Lin, Hsiao-Ju Chu, Shun-Fa Yang, Hsueh-Ju Lu

**Affiliations:** 1 Institute of Medicine, Chung Shan Medical University, Taichung, Taiwan.; 2 Department of Radiation Oncology, Changhua Christian Hospital, Changhua, Taiwan.; 3 School of Medicine, Chung Shan Medical University, Taichung, Taiwan.; 4 Department of Otolaryngology, Chung Shan Medical University Hospital, Taichung, Taiwan.; 5 Department of Otolaryngology, St. Martin De Porres Hospital, Chiayi, Taiwan.; 6 Department of Medical Research, Chung Shan Medical University Hospital, Taichung, Taiwan.; 7 Institute of Oral Sciences, Chung Shan Medical University, Taichung, Taiwan.; 8 Department of Dentistry, Chung Shan Medical University Hospital, Taichung, Taiwan.; 9 Division of Hematology and Oncology, Department of Internal Medicine, Chung Shan Medical University Hospital, Taichung, Taiwan.

**Keywords:** NSUN2, oral cavity squamous cell carcinoma, single-nucleotide polymorphism, susceptibility, clinicopathologic progression

## Abstract

NOP2/Sun RNA methyltransferase 2 (NSUN2), encoded by the *NSUN2* gene, is a nuclear RNA methyltransferase that catalyzes the methylation of cytosine to 5-methylcytosine (m5C). Although RNA modification has been widely discussed in cancer development and prognosis, the role of the *NSUN2* gene in oral cavity squamous cell carcinoma (OCSCC) is unclear. This was a retrospective, case-control study. A total of 2514 participants were enrolled, including 52.4% (1318/2514) diagnosed with OCSCC and others as health control. The impact of *NSUN2* rs4702373, rs166049, rs13181449, and rs8192120 on cancer development and prognosis were analyzed. Our results revealed that *NSUN2* rs13181449 allele TT was significantly associated with lower OCSCC risk, with an adjusted odd ratio (AOR) [95% confidence index (CI)] of 0.757[0.575-0.997]. For cigarette smokers, the impact of rs13181449 was more obvious that AOR [95% CI] of allele CT, TT, and CT+TT were 0.760 [0.583-0.991], 0.699 [0.493-0.990], 0.746 [0.580-0.960], respectively. For OCSCC patients, rs4702373 allele CT was independently associated with advanced histological grade. Expression levels between different allele mutations were various in the GTE database. Mutant rs4702373 and rs166049 were associated with higher expression than the wild type, conversely mutant rs13181449 had lower expression. In the TCGA database, the trend that patients with higher expression had worse survival than those with lower expression was shown. In conclusion, *NSUN2* rs13181449 was associated with lower cancer risk, especially for cigarette smokers. Unlikely other allele mutations, rs13181449 was correlated to lower expression. Patients with higher expression had worsened clinical outcomes.

## Introduction

Oral cavity squamous cell carcinoma (OCSCC) is one of the largest subpopulations of head and neck squamous cell carcinoma (HNSCC), the eighth most common cancer globally and the fourth most common cancer of males in Taiwan 1, 2. Although previous studies reported personal health habits impacted the risk of OCSCC development 3, genetic risks of OCSCC development were rarely discussed 4-8. Once the disease progresses to recurrent metastatic status, a poor prognostic situation 9, 10, genetic roles in prognosis also need to be clear. In the genomics era, genetic profiling associated with developmental risk and prognosis for OCSCC is significant.

Developing next-generation sequencing technologies improves genomic studies and treatment decisions in the modern era 11, 12. From the aspect of prevention medicine, some genetic variants also have been reported as significantly associated with cancer development, such as *BRCA1*/*BRCA2* mutations 13. The roles of single nucleotide polymorphisms were also discussed in the relationships of cancer risks across various cancer types 14, 15. Additionally, RNA modification is another critical issue in the occurrence and progression of cancer 16, 17. The mutation and regulation of long-noncoding RNA have been proven major role in cancer development for various cancer types 18-21. In previous study, the role of RNA modification, such as RNA methylation mediated by the *NSUN2* gene, was discussed, especially in cancer development and prognosis 22.

NOP2/Sun RNA methyltransferase 2 (NSUN2), a nuclear RNA methyltransferase that catalyzes the methylation of cytosine to 5-methylcytosine (m5C) at position 34 of intron-containing tRNA precursors, is a protein encoded by the *NSUN2* gene 23, 24. The physiologic function of NSUN2 is to regulate RNA modification, which is necessary to stabilize the anticodon-codon pairing and correctly translate the mRNA 24. NSUN2 directly plays an important role in tissue homeostasis, especially mitotic spindle stability. Other functions including cellular proliferation, migration, and differentiation were also presented 25. For malignancies, NSUN2 was discovered as a target of MYC and the depletion of NSUN2 wound impaired MYC-dependent proliferation 26. Prognostic roles of NSUN2 were also presented in various cancers, including gastric cancer, breast cancer, and others 27-29. In addition to the studies discussing the function of NSUN2 in malignancies, *NSUN2* polymorphisms have been reported to reduce cancer development in neuroblastoma 22. However, the roles of *NSUN2* polymorphisms as well as methyltransferase NSUN2 in OCSCC are limited.

To identify the impact of *NSUN2* polymorphisms on cancer development and prognosis, our study retrospectively analyzed their roles between patients with OCSCC and healthy people. Published databases, such as the Genotype-Tissue Expression (GTEx) Portal and TCGA, were used to validate our results.

## Materials and methods

### Study subjects

This retrospective case-control study included patients diagnosed with OCSCC, selected from the BioBank database of Chung Shan Medical University Hospital. The control group comprised healthy individuals aged 30 to 70 years, with normal cognitive function and no history of cancer, selected from the Taiwan Biobank. For the case group, individuals without a pathological diagnosis or those with second primary malignancies were excluded. Female participants were excluded from both the case and control groups, as nearly 90% of OCSCC patients were male 30. The study was approved by the Institutional Review Board of Chung Shan Medical University Hospital (CSMUH No: CS1-21151).

### Clinical data collection

Basic characteristics, including age, cigarette smoking, alcohol drinking, and betel quid chewing, were collected from the BioBank databases. Pathologic factors of the case group were also supported in our previous studies 31, 32. The American Joint Committee on Cancer's staging system (seventh edition) was used in this study 33. However, due to delinking and anonymity, cancer-specific clinical outcomes of the case group were limited and difficult to available.

### DNA extraction and genotyping

Genomic DNA was extracted from peripheral blood leukocytes by using QIAamp DNA blood mini kits (Qiagen, Valencia, CA, USA) according to the manufacturer's instructions. Because *NSUN2* polymorphisms, including rs4702373, rs166049, rs13181449, and rs8192120, were associated with cancer risk in previous studies 22, these polymorphisms were studied for OCSCC in our study. The results were further analyzed using SDS version 3.0. The details of DNA extraction and genotyping were published in our previous study 34.

### Published databases for validation

Several published databases were used to verify our results, including the Genotype-Tissue Expression (GTEx) portal and cBioPortal. The GTEx portal is a comprehensive public resource to study human gene expression and regulation, and its relationship to genetic variation across multiple diverse tissues and individuals, including whole-genome sequencing (WGS), whole-exome sequencing, and RNA-seq (https://gtexportal.org/home/) 35. The cBioPortal is an open-access, open-source resource for interactive exploration of multidimensional cancer genomics data sets, including the TCGA database (https://www.cbioportal.org/) 36.

### Statistical analysis

Clinicopathological parameters were compared using the *χ*^2^ and Fisher exact tests. Independent risks of cancer development were analyzed by using univariate and multivariate logistic regressions with the odd ratios (ORs) [95% confidence index (CI)]. Additionally, the impact of *NSUN2* polymorphisms in the OCSCC development was adjusted by age and personal health habits, with the so-called adjusted odd ratios (AOR). Significant association between genotypes and NSUN2 expression levels in the GTEx portal was detected with the linear regression model. Survival outcomes from the TCGA database were analyzed using Kaplan-Meier plots and the log-rank test. A two-sided *P* value of <0.05 was statistically significant. SPSS (version 21.0, SPSS Inc., Chicago, IL, USA) was used for all statistical analyses.

## Results

### Baseline characteristics

A total of 2514 participants were enrolled in the study, including 52.4% (1318/2514) in the case group and others (47.6%, 1196/2514) in the control group. The case group, diagnosed with OCSCC, were elderly, with more cigarette smoking, alcohol drinking, and betel quid chewing than the control group (all *P* < 0.001). Basic characteristics are shown in Table 1.

### *NSUN2* rs13181449 prevents the development of OCSCC

The distributions of all allele mutations were shown. Among all participants, the allele mutant frequencies of *NSUN2* rs4702373, rs166049, rs13181449, and rs8192120 were 32.4% (815/2514), 35.6% (895/2514), 67.3% (1692/2514), and 50.9% (1280/2514), respectively. The distributions of the allele mutations between the case and control groups were similar, with *P* values of 0.312, 0.872, 0.348, and 0.360, respectively. To know the impacts of *NSUN2* polymorphisms on the development of OCSCC, logistic regression analysis was done to identify the odd ratio (95% confidence index [CI]) of each allele. After adjusting to age, cigarette smoking, alcohol drinking, and betel quid chewing, the participants with rs13181449 allele TT significantly had lower OCSCC development than those without (AOR [95% CI], 0.757[0.575-0.997]) (Table 2).

For cigarette smokers, one of the most common subgroups representing 65.4% (1643/2514) of all participants, rs13181449 allele mutations were also significantly associated with low OCSCC development. AORs [95% CI] of rs13181449 allele CT, TT, and CT+TT were 0.760 [0.583-0.991], 0.699 [0.493-0.990], 0.746 [0.580-0.960], respectively. *NSUN2* rs13181449 decreasing OCSCC risk was advanced for cigarette smokers (Table 2).

### *NSUN2* mRNA expression among different tissues

**Figure 1:**
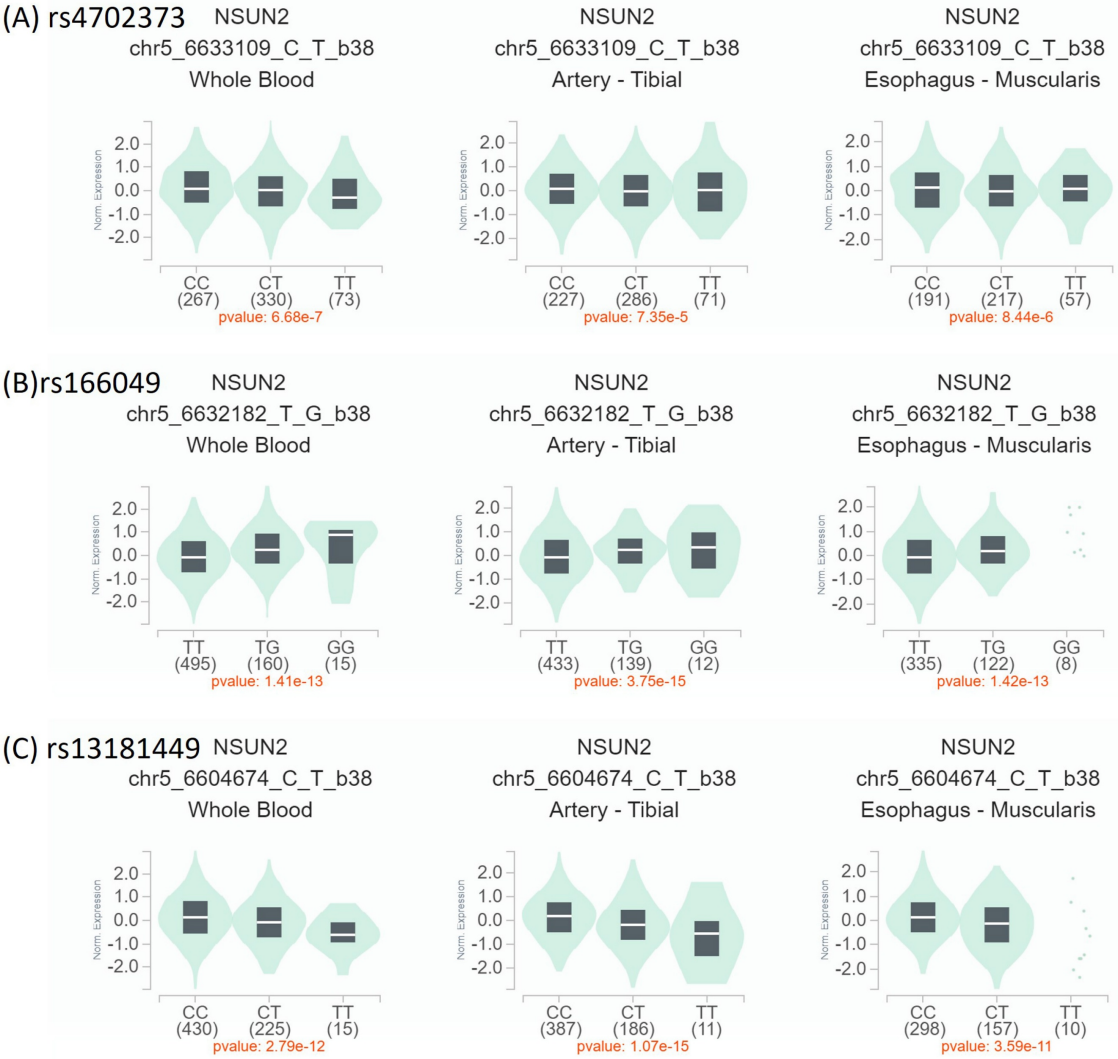
***NSUN2* expression by Genotype-Tissue Expression (GTEx) Portal (https://www.gtexportal.org/home/).** The expression levels of individual NSUN2 polymorphisms. Expression quantitative trait loci (eQTL) violin plots between mutant- and wild-type polymorphisms were compared in whole blood, artery-tibial, and esophagus muscularis. (A, B) The participants with mutant rs4702373 and rs166049 were associated with higher expression than those with wild type. (C) Conversely, those with mutant rs13181449 had lower expression than others with wild type.

To verify our finding, the expressions of *NSUN2* allele mutations among different tissues were compared based on the GTEx database. The participants with rs4702373 and rs166049 mutations were significantly associated with higher *NSUN2* expression in whole blood, artery, and upper aerodigestive soft tissue (esophageal muscularis) than those without (all *P* values < 0.05). However, the participants with rs13181449 mutation had lower expression than others (*P* < 0.05) (Figure 1). It might indirectly support that the participants with lower *NSUN2* expression levels were related to lower OCSCC development. Future warranted studies were needed.

### Prognostic role of *NSUN2* polymorphisms in OCSCC

The prognostic roles of *NSUN2* allele mutations in OCSCC were also studied. Among the participants diagnosed with OCSCC, allele mutations of rs4702373, rs166049, rs13181449, and rs8192120 were 33.5% (442/1318), 35.9% (473/1318), 66.5% (877/1318), and 52.2% (668/1318), respectively. Basic characteristics between the OCSCC patients with and without allele mutations were insignificant differences, except for the patients with rs4702373 allele mutation who were associated with advanced histological grade than those without (*P* = 0.038) (Table 3). However, for the patients with cigarette smoking, the patients with rs4702373 mutation also had a trend of advanced histological grade than those without but insignificant in statistic (advanced histologic grade, patients with and without rs4702373 mutation, 85.5% vs. 81.3%, respectively,* P* = 0.058) (Table 4).

Independent roles of *NSUN2* alleles for histologic grades were analyzed by using univariant and multivariate logistic regression. In Table 5, cigarette smoking, betel quid chewing, pathologic N staging, and rs4702373 allele CT were significantly associated with moderate-to-poor histological grade in univariant analysis. But only rs4702373 allele CT was independent impacting histological grade (OR [95% CI], 1.451[1.017-2.069]). Other allele mutations were not associated with the impact of histological grade formation in analyses.

### Relationship between *NSUN2* expression and clinical outcomes

In addition, the TCGA database was also used for validation. Because nearly one-third of OCSCC patients in our cohort had rs4702373 allele mutation and the mutant allele might be associated with higher *NSUN2* expression, a total of 515 HNSCC patients from the TCGA database were divided into the two groups of high- (33.4%, 172/515) and low- (66.6%, 343/515) *NSUN2* expression. The high-NSUN2 group significantly worsened in overall survival and disease-free survival, with *P* values of 0.031 and 0.030, respectively. The impact of *NSUN2* expression was also presented in both patients with and without human papillomavirus (HPV) associated. Although there were insignificant differences in overall survival and disease-free survival between the patients with high- and low- *NSUN2* expression, either with or without HPV associated, the trend showed that the patients with high expression seemed worse survivals than those with low expression.

## Discussion

A total of 2514 participants were retrospectively enrolled in our study, with 52.4% belonging to the case group diagnosed with OCSCC and others as healthy control. *NSUN2* rs13181449 allele TT was significantly associated with a lower OCSCC development. For cigarette smokers, the effect of *NSUN2* rs13181449 decreasing the risk of OCSCC development was more obvious. In the prognostic role, the *NSUN2* rs4702373 CT allele was an independent factor associated with the formation of advanced histological grade for the patients diagnosed with OCSCC. The published database validated that except for rs13181449 mutations associated with lower expression levels, the participants with *NSUN2* polymorphism mutations significantly had higher expression than those without. For both subgroups with or without HPV associated, the patients with higher expression levels had worse clinical outcomes than those with lower expression. Future studies were needed.

There were several advantages in our study as follows. First, this was a large real-world population, with a total of 2514 participants, and 52.4% of them diagnosed with OCSCC. It was representative to discuss the role of *NSUN2* in OCSCC. In addition, although genomic studies were important in the modern era, genetic roles in decreasing the risk of malignancies were limited 13. In our study, *NSUN2* rs13181449 independently reduced the risk of OCSCC development, especially for the subgroup with cigarette smoking. Third, *NSUN2* expressions were various in several cancer types. In OCSCC, the patients with higher *NSUN2* expression were associated with worse prognosis than those without. However, different allele mutations might be associated with opposite gene expressions. Future studies to validate this issue should be careful.

DNA methylation has been reported as an important regulator of gene transcription, and its carcinogenetic role is also an interesting issue in the last few years. Alterations in DNA methylation are common in a variety of tumors as well as in development 37. Recently, the roles of RNA methylation have been discussed widely 38, 39. The Biological significance of NSUN2 upregulating cell proliferation and metastasis was reported, and the TCGA database also showed that in tumor specimens, *NSUN2* expressions are higher than those in normal tissue across several cancer types 25, 40, 41. For HNSCC, Lu et al. study presented that *NSUN2* expression was 1.99-fold upregulation versus normal tissue. The patients associated with higher *NSUN2* were correlated with worse survival than those with lower 42. *NSUN2* expression was also reported as a potential biomarker that immune checkpoint therapy might benefit patients with higher expression and less effective for those with lower expression 43. Our study reported that for OCSCC, patients with higher* NSUN2* expression were associated with worsened survival. In addition, *NSUN2* rs4702373 allele mutation, related to higher expression, was independent of advanced histological grade. This finding was correlative to previous studies. However, the expression levels might vary between different alleles. Compared with normal tissue, the expression level of the rs13181449 mutation was lower, and conversely, the expression level of the rs4702373 and rs166049 mutations was higher. This point needed to be advanced validated.

Although the mechanism that rs13181449 associated with lower expression needed to be studied, patients with rs13181449 mutation seemed to have a lower risk of cancer development. In Lin et al. study, *NSUN2* rs13181449 was significantly associated with lower neuroblastoma risk (CT/TT vs. CC: AOR [95% CI]: 0.69 [0.50-0.97]) 22. In our cohort, *NSUN2* rs13181449 also reduced the development of OCSCC, especially for cigarette smokers. Although detailed mechanisms between *NSUN2* mutation, cigarette smokers, and cancer development are unknown, it might be because cigarette smoking might increase HIF-1-alpha levels, which mediated RNA modification through complex regulation, such as cell cycle, PI3K-AKT pathway, and others 44-46. Therefore, *NSUN2* rs13181449-associated lower expression might decrease the activation of the cell cycle and PI3K-AKT pathway, protecting cancer development more obviously for patients with cigarette smoking. Advanced experiment studies are needed.

Several limitations were presented in this study. Although this was a large cohort study, with more than two thousand five hundred participants enrolled, functional experiments *in vitro* and *in vivo* were needed to validate. In addition, the anonymous and delinked policy due to ethical issues caused detailed clinicopathological information not available. This information was also important for validation. Third, expression levels of different *NSUN2* polymorphisms were various in the published database. Detailed mechanisms were suggested to support our findings, especially for the relationship between *NSUN2* rs13181449, cigarette smokers, and cancer development.

In conclusion, the roles of *NSUN2* polymorphisms in OCSCC development and prognosis were rarely discussed. In our study, *NSUN2* rs13181449 was independent in decreasing the risk of OCSCC, especially for cigarette smokers. However, based on the published database, expression levels of different polymorphisms might be various. In the prognostic role, the patients with higher expression were associated with advanced histological grade and worsened survival. *In vitro* and *in vivo* advanced functional studies were needed to validate the results.

## Funding

This study was supported by Chung Shan Medical University Hospital (CSH-2023-C-006).

## Figures and Tables

**Figure 2:**
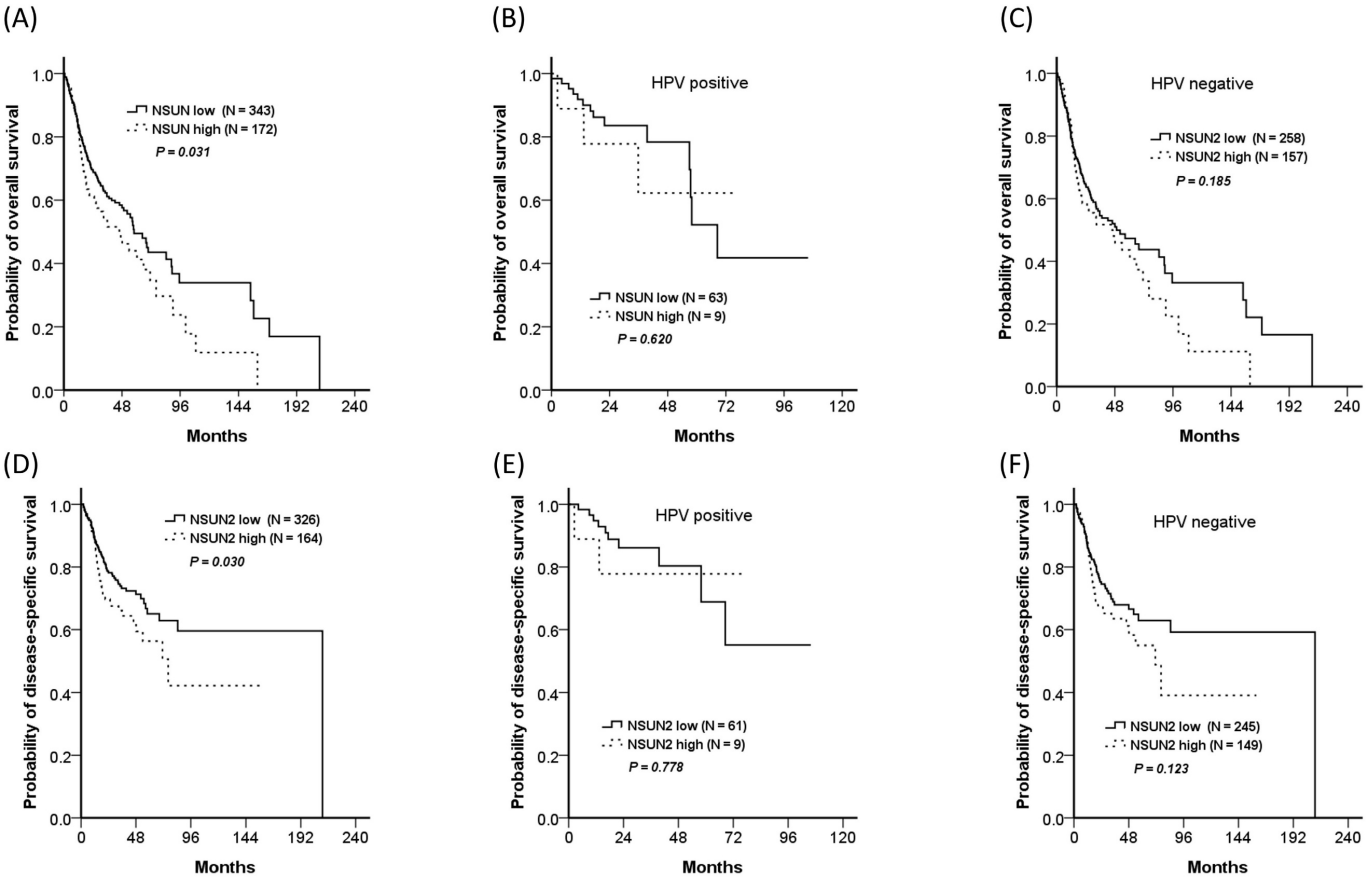
** Overall survivals of the high- and low-*NSUN2* HNSCC patients from the TCGA database.** The trend showed the high *NSUN2* group had worsened survival than the low *NSUN2* group. (A) For all patients, 5-year overall survivals were 44.0% and 49.5% for the high- and low-*NSUN2* groups, respectively (*P* = 0.031), (B) 62.2% and 52.2% for HPV positive population (*P* = 0.620), (C) 43.6% and 47.3% for HPV negative population (*P* = 0.185). (D) For all patients, 5-year disease-specific survivals were 56.3% and 65.1% (*P* = 0.030), (E) 77.8% and 68.9% for HPV positive population (*P* = 0.778), (F) 55.0%% and 62.9% for HPV negative population (*P* = 0.123).

**Table 1:** Basic characteristics of the patients with oral cancer and healthy controls

Variable	Patients (N=1318)	Controls (N=1196)	*P* value
Age (yrs)			0.002
≥ 55	771 (58.5%)	630 (52.7%)	
<55	547 (41.5%)	566 (47.3%)	
Cigarette smoking			<0.001
Yes	1008 (76.5%)	635 (53.1%)	
No	310 (23.5%)	561 (46.9%)	
Alcohol drinking			<0.001
Yes	497 (37.7%)	236 (19.7%)	
No	821 (62.3%)	960 (80.3%)	
Betel quid chewing			<0.001
Yes	878 (66.6%)	198 (16.6%)	
No	440 (33.4%)	998 (83.4%)	
Pathologic staging			NA
I+II	603 (45.8%)		
III+IV	715 (54.2%)		
Pathologic T staging			NA
T1+T2	634 (48.1%)		
T3+T4	684 (51.9%)		
Pathologic N staging			NA
N0	900 (68.3%)		
N+	418 (31.7%)		
Pathologic M staging			NA
M0	1312 (99.5%)		
M1	6 (0.5%)		
Histological differentiation			NA
Well	204 (15.5%)		
Moderate to poor	1114 (84.5%)		

**Table 2:** Odds ratios (OR) and 95% confidence interval (CI) of oral cancer associated with *NSUN2* genotypic frequencies.

Variable	Patients (N, %)	Controls (N, %)		OR (95% CI)	AOR (95% CI)^ a^
All participants					
	N = 1318	N = 1196	*P* value		
**rs4702373**			0.312		
CC	876(66.5%)	823(68.8%)		1	1
CT	391(29.7%)	337(28.2%)		1.090(0.916-1.297)	1.111(0.907-1.361)
TT	51(3.9%)	36(3.0%)		1.331(0.860-2.061)	1.359(0.825-2.238)
CT+TT	442(33.5%)	373(31.2%)		1.113(0.942-1.316)	1.136(0.935-1.381)
**rs166049**			0.872		
TT	845(64.1%)	774(64.7%)		1	1
TG	419(31.8%)	370(30.9%)		1.037(0.875-1.230)	1.041(0.854-1.269)
GG	54(4.1%)	52(4.3%)		0.951(0.642-1.409)	0.922(0.579-1.467)
TG+GG	473(35.9%)	422(35.3%)		1.027(0.872-1.209)	1.027(0.849-1.242)
**rs13181449**			0.348		
CC	441(33.5%)	381(31.9%)		1	1
CT	659(50.0%)	592(49.5%)		0.962(0.806-1.147)	0.908(0.740-1.115)
TT	218(16.5%)	223(18.6%)		0.845(0.670-1.065)	**0.757(0.575-0.997)**
CT+TT	877(66.5%)	815(68.1%)		0.930(0.787-1.099)	0.870(0.716-1.057)
**rs8192120**			0.360		
CC	630(47.8%)	604(50.5%)		1	1
CA	584(44.3%)	497(41.6%)		1.127(0.957-1.327)	1.153(0.953-1.395)
AA	104(7.9%)	95(7.9%)		1.050(0.778-1.416)	1.047(0.736-1.489)
CA+AA	688(52.2%)	592(49.5%)		1.114(0.953-1.303)	1.135(0.946-1.362)
Cigarette smoking					
	N = 1008	N = 635			
**rs4702373**			0.448		
CC	664(65.9%)	437(68.8%)		1	1
CT	303(30.1%)	176(27.7%)		1.133(0.908-1.414)	1.138(0.878-1.474)
TT	41(4.1%)	22(3.5%)		1.227(0.721-2.088)	1.295(0.705-2.380)
CT+TT	344 (34.1%)	198 (31.2%)		1.143(0.925-1.414)	1.157(0.902-1.483)
**rs166049**			0.760		
TT	647(64.2%)	406(63.9%)		1	1
TG	318(31.5%)	197(31.0%)		1.013(0.816-1.258)	0.984(0.764-1.267)
GG	43(4.3%)	32(5.0%)		0.843(0.525-1.355)	0.861(0.490-1.512)
TG+GG	361 (35.8%)	229 (36.1%)		0.989(0.804-1.217)	0.965(0.758-1.230)
**rs13181449**			0.154		
CC	341(33.8%)	186(29.3%)		1	1
CT	499(49.5%)	333(52.4%)		0.817(0.652-1.025)	**0.760(0.583-0.991)**
TT	168(16.7%)	116(18.3%)		0.790(0.587-1.063)	**0.699(0.493-0.990)**
CT+TT	667 (66.2%)	449 (70.7%)		0.810(0.654-1.005)	**0.746(0.580-0.960*)***
**rs8192120**			0.960		
CC	486(48.2%)	310(48.8%)		1	1
CA	438(43.5%)	274(43.1%)		1.020(0.829-1.255)	1.041(0.817-1.328)
AA	84(8.3%)	51(8.0%)		1.051(0.721-1.530)	1.098(0.707-1.704)
CA+AA	522 (51.8%)	325 (51.2%)		1.025(0.840-1.250)	1.050(0.832-1.324)

^a^ Adjusted for the effects of age, cigarette smoking, alcohol drinking, and betel quid chewing

**Table 3:** The distributions of demographical characteristics of *NSUN2* allele mutation in all OCSCC patients (N = 1318)

	rs4702373			rs166049			rs13181449			rs8192120		
Variable	CT+TT N = 442 (%)	CC N = 876 (%)	P value	TG+GG N = 476 (%)	TT N = 845 (%)	P value	CT+TT N = 887 (%)	CC N = 441 (%)	P value	CA+AA N = 688 (%)	CC N = 630 (%)	P value
**Age >= 55**	260 (58.8)	551 (58.3)	0.456	286(60.5)	485(57.4)	0.152	510(58.2)	261(59.2)	0.383	403(58.6)	368(58.4)	0.498
**Personal history**												
Cigarette smoking	344(77.8)	664(75.8)	0.227	361(76.3)	647(76.6)	0.485	667(76.1)	341(77.3)	0.330	522(75.9)	486(77.1)	0.316
Alcohol drinking	174(39.4)	323(36.9)	0.205	183(38.7)	314(37.2)	0.312	335(38.2)	162(36.7)	0.324	250(36.3)	247(39.2)	0.155
Betel quid chewing	299(67.6)	579(66.1)	0.308	308(65.1)	570(67.5)	0.211	587(66.9)	291(66.0)	0.388	454(66.0)	424(67.3)	0.328
**Pathologic staging**			0.513			0.450			0.396			0.057
Stage I+II	202(45.7)	401(45.8)		218(46.1)	385(45.6)		404(46.1)	199(45.1)		300(43.6)	303(48.1)	
Stage III+IV	240(54.3)	475(54.2)		255(53.9)	460(54.4)		473(53.9)	242(54.9)		388(56.4)	327(51.9)	
**Pathologic T staging**			0.325			0.284			0.347			0.124
T1/2	217(49.1)	417(47.6)		233(49.3)	401(47.5)		418(47.7)	216(49.0)		320(46.5)	314(49.8)	
T3/4	225(50.9)	459(52.4)		240(50.7)	444(52.5)		459(52.3)	225(51.0)		368(53.5)	316(50.2)	
**Pathologic N staging**			0.293			0.379			0.323			0.163
N0	297(67.2)	603(68.8)		326(68.9)	574(67.9)		603(68.8)	297(67.3)		461(67.0)	439(69.7)	
N+	145(32.8)	273(31.2)		147(31.1)	271(32.1)		274(31.2)	144(32.7)		227(33.0)	191(30.3)	
**Metastasis**			0.677			0.371			0.678			0.614
M0	440(99.5)	872(99.5)		470(99.4)	842(99.6)		873(99.5)	439(99.5)		685(99.6)	627(99.5)	
M1	2(0.5)	4(0.5)		3(0.6)	3(0.4)		4(0.5)	2(0.5)		3(0.4)	3(0.5)	
**Cell differentiation grade**			**0.038**			0.247			0.418			0.271
Well	57(12.9)	147(16.8)		78(16.5)	126(14.9)		134(15.3)	70(15.9)		102(14.8)	102(16.2)	
Moderate or poor	385(87.1)	729(83.2)		395(83.5)	719(85.1)		743(84.7)	371(84.1)		586(85.2)	528(83.8)	

**Table 4:** The distributions of demographical characteristics of *NSUN2* allele mutation in OCSCC patients with Cigarette smoking (N = 1008)

	rs4702373			rs166049			rs13181449			rs8192120		
Variable	CT+TT N = 344 (%)	CC N = 664 (%)	P value	TG+GG N = 361 (%)	TT N = 647 (%)	P value	CT+TT N = 667 (%)	CC N = 341 (%)	P value	CA+AA N = 522 (%)	CC N = 486 (%)	P value
**Age >= 55**	191(55.5)	370(55.7)	0.502	212(58.7)	349(53.9)	0.081	368(55.2)	193(56.6)	0.358	290(55.6)	27(55.8)	0.499
**Personal history**												
Cigarette smoking	NA	NA		NA	NA		NA	NA		NA	NA	
Alcohol drinking	160(46.5)	293(44.1)	0.256	170(47.1)	283(43.7)	0.169	303(45.4)	150(44.0)	0.357	223(42.7)	230(47.3)	0.080
Betel quid chewing	283(82.3)	531(80.0)	0.214	286(79.2)	528(81.6)	0.201	544(81.6)	270(79.2)	0.205	421(80.7)	393(80.9)	0.498
**Pathologic staging**			0.288			0.256			0.405			**0.035**
Stage I+II	154(44.8)	311(46.8)		172(47.6)	293(45.3)		310(46.5)	155(45.5)		226(43.3)	239(49.2)	
Stage III+IV	190(55.2)	353(53.2)		189(52.4)	354(54.7)		357(53.5)	186(54.5)		296(56.7)	247(50.8)	
**Pathologic T staging**			0.419			0.389			0.397			0.145
T1/2	172(50.0)	326(49.1)		181(50.1)	317(49.0)		332(49.8)	166(48.7)		249(47.7)	249(51.2)	
T3/4	172(50.0)	338(50.9)		180(49.9)	330(51.0)		335(50.2)	175(51.3)		273(52.3)	237(48.8)	
**Pathologic N staging**			0.407			0.350			0.409			0.130
N0	233(67.7)	456(68.7)		250(69.3)	439(67.9)		458(68.7)	231(67.7)		348(66.7)	341(70.2)	
N+	111(31.6)	208(31.3)		111(30.7)	208(32.1)		209(31.3)	110(32.3)		174(33.3)	145(29.8)	
**Metastasis**			0.445			0.588			0.451			0.466
M0	343(99.7)	660(99.4)		359(99.4)	644(99.5)		663(99.4)	340(99.7)		520(99.6)	483(99.4)	
M1	1(0.3)	4(0.6)		2(0.6)	3(0.5)		4(0.6)	1(0.3)		2(0.4)	3(0.6)	
**Cell differentiated grade**			0.058			0.183			0.523			0.274
Well	50(14.5)	124(18.7)		68(18.8)	106(16.4)		115(17.2)	59(17.3)		86(16.5)	88(18.1)	
Moderate or poor	294(85.5)	540(81.3)		293(81.2)	541(83.6)		554(82.8)	282(82.7)		436(83.5)	398(81.9)	

**Table 5:** Univariate and multivariate logistic regression for moderate to poor histologic differentiation in all oral cancer patients

	All patients	
	Univariate	Multivariate
Variable	OR (95% CI), P value	OR (95% CI), P value
Age (yrs)		
≥ 55 vs. <55	0.872(0.643-1.184), 0.382	
Personal history		
Cigarette smoking (yes vs. no)	0.514(0.341-0.774), 0.001	0.665(0.411-1.078)
Alcohol drinking (yes vs. no)	0.862(0.636-1.169), 0.340	
Betel quid chewing (yes vs. no)	0.567(0.401-0.801), 0.001	0.682(0.451-1.032)
Pathologic T staging		
pT3/4 vs. pT1/2	1.146(0.850-1.545), 0.371	
Pathologic N staging		
pN+ vs. pN0	2.737(1.848-4.052), <0.001	2.594(1.744-3.857), <0.001
Metastasis		
M1 vs. M0	0.364(0.066-2.000), 0.245	
**rs4702373**		
CC	Reference	Reference
CT	**1.441(1.015-2.045), 0.041**	**1.451(1.017-2.069), 0.040**
TT	0.941(0.488-1.975), 0.872	
CT+TT	1.362(0.979-1.894), 0.066	
**rs166049**		
TT	Reference	
TG	0.831(0.606-1.139), 0.831	
GG	1.717(0.671-4.394), 0.259	
TG+GG	0.887(0.652-1.207), 0.447	
**rs13181449**		
CC	Reference	
CT	1.134(0.810-1.587), 0.463	
TT	0.840(0.547-1.288), 0.423	
CT+TT	1.046(0.764-1.433), 0.779	
**rs8192120**		
CC	Reference	
CA	1.183(0.863-1.621), 0.297	
AA	0.811(0.477-1.381), 0.441	
CA+AA	1.110(0.823-1.496), 0.494	
